# Corps étranger inhabituel intra-urétral : à propos d’un cas

**DOI:** 10.11604/pamj.2017.27.58.12644

**Published:** 2017-05-26

**Authors:** Hicham El Bote, Ernest Hage, Rami Fares

**Affiliations:** 1 Service d’Urologie A, CHU Ibn Sina, Rabat, Maroc; 2 Service d’Urologie, Centre Hospitalier de Soissons, Soissons, France

**Keywords:** Corps étranger, urètre, traitement endoscopique, Foreign body, urethra, endoscopic treatment

## Abstract

De nombreux articles de la littérature médicale rapportent des cas rares d'introduction de corps étrangers dans l'urètre. Ils sont souvent secondaires, soit à une curiosité érotique, soit à des troubles psychiatriques, ou rarement lors d'une tentative d'évacuation des urines. Le diagnostic reste clinique aidé par l'imagerie. Le traitement est le plus souvent endoscopique, mais dans certains cas le recours à la chirurgie ouverte est nécessaire. L'approche thérapeutique doit être systématiquement complétée par une évaluation psychiatrique du malade. Nous rapportons le cas d'un homme de 64 ans aux antécédents de troubles du comportement, il s'est introduit un fil électrique dans l'urètre. La prise en charge constituait en une extraction endoscopique sous anesthésie locorégionale. A sa sortie le patient a été référé en consultation psychiatrique.

## Abstract (en)

In medical literature there are several case reports of foreign bodies introduced into the urethra. Foreign bodies are usually inserted as result of erotic activities, psychiatric disorders and, rarely, in an attempt to evacuate the urine. Diagnosis is based on clinical examination and imaging tests. Endoscopic management is the treatment of choice but, in some cases, open surgery may be necessary. The therapeutic approach should be systematically complemented by a psychiatric assessment of the patient. We report the case a 64-year old man with a personal history of behavioral disorders who introduced an electric wire in his urethra. The patient underwent endoscopic extraction under regional anesthesia. The patient at the time of discharge was referred to a psychiatrist.

## Introduction

L'auto-insertion de corps étrangers dans l'urètre est une situation inhabituelle, survenant souvent lors des impulsions érotiques y compris la masturbation ou d´autres formes de déviation sexuelle, ou au cours de certaines maladies psychiatriques qui représentent les causes les plus fréquentes et les plus graves. Malgré que la symptomatologie est polymorphe le diagnostic clinique est généralement facile aidé par l'imagerie. L'extraction endoscopique reste le traitement de référence, mais le recours à la chirurgie ouverte est parfois nécessaire. L'approche thérapeutique doit être systématiquement complétée par une évaluation psychiatrique du malade.

## Patient et observation

**Figure 1:**
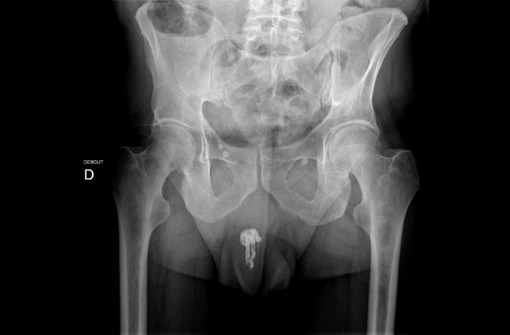
Aspect du corps étranger à la radiographie standard du bassin

**Figure 2:**
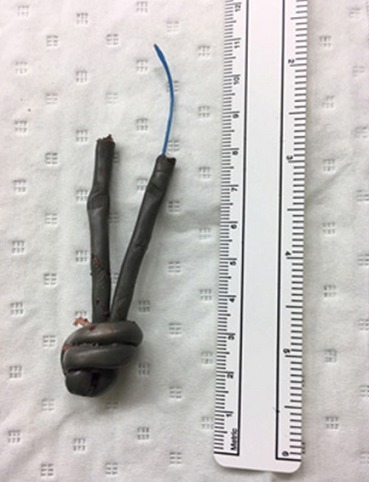
Intégralité du fil électrique après son extraction endoscopique

Il s'agissait de Mr B.J âgé de 64 ans, marié, reçu au service d'accueil des urgences pour un corps étranger urétral (fil électrique) ; introduit depuis 4 jours dans le but d'évacuer le contenu vésical selon le patient. Il n'aurait pas d'antécédents médico-chirurgicaux pathologiques connues mais l'anamnèse retrouve des antécédents de troubles du comportement avec notion d'introduction de corps étranger dans le rectum il y'a quatre ans. A l'examen le patient était en bon état général avec une conscience claire. Une masse périnéale dure était palpée sans notion d'urétrorragie ni d'hématurie. La miction urinaire se faisait par goutte-à-goutte. La radiographie standard du bassin a objectivé une opacité de tonalité métallique qui correspondait au câble électrique avec un nœud visible (Figure 1). Après le bilan préopératoire, un traitement mini-invasif par voie endoscopique sous anesthésie locorégionale et en position de la taille a permis l'extraction du corps étranger (Figure 2). Les suites opératoires ont été simples. Le patient a été référé en consultation psychiatrique.

## Discussion

De nombreux articles médicaux rapportent des cas d'auto-insertion de corps étrangers dans l'urètre. La majorité de ces situations sont rencontrés lors des impulsions érotiques y compris la masturbation ou d´autres formes de déviation sexuelle, ou au cours de certaines maladies psychiatriques dans un but d'automutilation, et qui représentent les causes les plus fréquentes et les plus graves [1]. Les corps étrangers décrits dans la littérature sont très variés: pince à épiler, épingle à cheveux, punaise, caillou, trombone et câble électrique comme dans le cas inhabituel de notre patient [2]. Habituellement le malade se présente aux urgences pour dysurie, douleur périnéale, urétrorragie, voire une hématurie. Les complications au stade chronique sont à type de rétention urinaire, d'infection urinaire, de septicémie, de lithiases urinaires, de sténose de l'urètre [3], et rarement de nécrose du pénis [4]. Le diagnostic est aisément posé par l'examen clinique, et les examens complémentaires radiologiques (ASP, l'échographie ou la TDM) sont utiles surtout pour préciser la taille, la forme, la localisation et l'orientation du corps étranger, afin d'aider au choix thérapeutique. L'extraction du corps étranger par voie endoscopique reste le traitement mini-invasif de référence [5], mais dans certains cas plus complexes le recours à la chirurgie ouverte est nécessaire [6]. L'évaluation psychiatrique doit être systématique pour ne pas passer à côté d´un trouble psychiatrique grave qui risque d'engager la responsabilité juridique de l´urologue et le pronostic vital du malade s'il est non diagnostiqué [7].

## Conclusion

Vu que la plupart des corps étrangers intra-urétraux sont insérés au cours d'une masturbation, et le plus souvent dans un contexte de maladie mentale avec souvent des actes impulsifs autoagressifs. Le traitement doit comporter systématiquement deux volets, d´une part, l´extraction du corps étranger de préférence par voie endoscopique, et d´autre part une évaluation psychiatrique avec une prise en charge thérapeutique adaptée.

## Conflits d’intérêts

Les auteurs ne déclarent aucun conflit d´intérêt.
